# Abnormal balance control mechanisms during dynamic reaching forward and quiet standing in patients with anterior cruciate ligament reconstruction

**DOI:** 10.3389/fphys.2023.1176222

**Published:** 2023-07-14

**Authors:** Wei Wang, Xudong Li, Runxiu Shi, Cheng Wang, Ke Zhang, Xiaomin Ren, Hui Wei

**Affiliations:** ^1^ Department of Physical Medicine and Rehabilitation, Qilu Hospital of Shandong University, Jinan, Shandong, China; ^2^ Department of Orthopedics, Qilu Hospital, Shandong University, Jinan, Shandong, China

**Keywords:** ACL reconstruction, balance control mechanism, center of pressure, reaching forward, rehabilitation

## Abstract

**Purpose:** Postural instability and decreased balance control ability have been observed in patients after anterior cruciate ligament (ACL) reconstruction. Herein, we examined the abnormal balance control mechanisms of these patients during dynamic reaching forward and quiet standing, providing a quantitative index for rehabilitation assessment.

**Methods:** We enrolled ACL reconstruction patients 6–8 months after surgery, and 14 gender- and age-matched healthy volunteers. The IKDC and Lysholm were applied in each patient after ACL reconstruction. All participants conducted the quiet standing and reaching forward (RF) tests at the specified locations on force plates. The ground reaction force, center of pressure (COP), and kinematics signals were recorded. The maximal reach distance (MRD), speed of RF, length of COP, peak speed of COP in anterior-posterior direction (AP-COP), and weight bearing ratio (WBR) of the affected limb were calculated in the RF test. The COP speed, COP amplitude, frequency components of COP and WBR were extracted during quiet standing.

**Results:** We observed that the speed of RF in the patients after ACL reconstruction was significantly lower than that of controls (*p* < 0.05). The COP length during RF was positively correlated with the Lysholm scale in the affected limb of patients (r = 0.604, *p* < 0.05). The peak of AP-COP speed during RF in the affected limb of patients was significantly lower than that of the healthy controls (*p* < 0.05), and positively correlated with the IKDC scale (r = 0.651, *p* < 0.05). WBR on the affected limb of patients during RF were significantly lower than that of controls (*p* < 0.05). The mean (r = −0.633, *p* < 0.05) and peak (r = −0.643, *p* < 0.05) speeds of COP during quiet standing were negatively correlated with the IKDC scale value. The amplitude of AP-COP on the contralateral side of patients was significantly higher than that of controls during quiet standing (*p* < 0.05).

**Conclusion:** Patients after ACL reconstruction performed decreased postural control capacity, especially in dynamic balance, and were accompanied by deficiencies in proprioception. The COP length, peak speed of COP during RF and COP speed during quiet standing could be considered as quantitative index of balance function assessment after ACL reconstruction.

## 1 Introduction

Stable posture control for quiet standing and movement is a fundamental motor function during daily activities. During convalescence following anterior cruciate ligament (ACL) reconstruction, patients performed imperfect motor function, such as postural instability, abnormal gait, and difficulty in sit-stand transfer (Clark et al., 2014; Difabio et al., 2018; Hoch et al., 2018). The balance control capacity is the foundation of various motor functions. Evaluating the postural stability for balance control during dynamic and static conditions may provide quantifications for muscular disorders and would aid in the development of strategies to improve the balance capacity of patients with ACL reconstruction during rehabilitation (Kim et al., 2022).

Compared to these technologies for postural stability analysis, such as subjective assessment, limb vibrations, trunk force line, and spatial kinematics, the center of pressure (COP) is relatively easy to record, is linked to postural control, and indicates critical information regarding the muscular coordination in lower-limb (Ty Hopkins, 2014; Kilby et al., 2016). In a previous study, patients with ACL reconstruction showed increased amplitude variability and speed value of COP compared to healthy individuals, reflecting a decreased balance control capacity and aggravated postural sway during standing (Clark et al., 2014).

The reaching forward (RF) test during standing has previously been applied to evaluate balance capacity clinically. A previous study reported that the measurement of the maximal distance during reaching forward horizontally with arms outstretching can be considered as a reliable parameter to evaluate the balance capacity in dynamic condition (Bohannon et al., 2020). In the RF test, greater maximal reach distance (MRD) indicates larger stability boundary and lower risk of falls in daily movement. Indeed, it has been reported in previous study that patients with low back pain showed decreased MRD and muscle flexibility during RF (Johnson & Thomas, 2010). The COP antedisplacement can be used to indicate the security boundary during postural adjustment and reflects the movement of muscles to control the rotation of ankle (Gandolfi et al., 2021).

The insufficient balance function could be observed in patients with ACL reconstruction. However, it remains unknown that the changes of COP characteristics and the relationship between these characteristics and knee function during RF 6 months after ACL reconstruction. This study aimed to investigate the dynamic and static balance control capacity of patients with ACL reconstruction. We therefore determined whether patients with ACL reconstruction show balance disorders by exploring the MRD values, COP antedisplacement, peak speed of COP in anterior-posterior direction (AP-COP) during RF, amplitude variability of COP, and speed of COP trajectory during quiet standing. This study explored the abnormal postural control pattern and could provide quantitative assessment indexes for rehabilitation after ACL reconstruction.

## 2 Methods

### 2.1 Participants

We enrolled 14 patients who had undergone ACL reconstruction 6–8 months prior and formal rehabilitation training, as well as 14 healthy volunteers as controls. The sample size was determined by power analysis. Patients were recruited from the Qilu Hospital of Shandong University, from 14 August 2021 to 21 June 2022. All patients (aged 18–60 years old) had been clinically diagnosed with unilateral ACL injury, had undergone surgery 6–8 months prior, and could complete all tasks in the experiment independently. Individuals with a history of cardiovascular and cerebrovascular diseases, muscle or bone injuries in their low back or lower extremities, vertigo or vestibular disease, severe visual defects, or cognitive difficulties were excluded. The operator explained the purpose and potential risks of the experiment in detail to every subject. All subjects signed an informed consent form. The protocols of the current study had been approved by the medical ethics committee of Qilu Hospital of Shandong University (KYLL-202107-041). The study was conducted in accordance with relevant regulations and guidelines.

### 2.2 Experimental set-up

**FIGURE 1:**
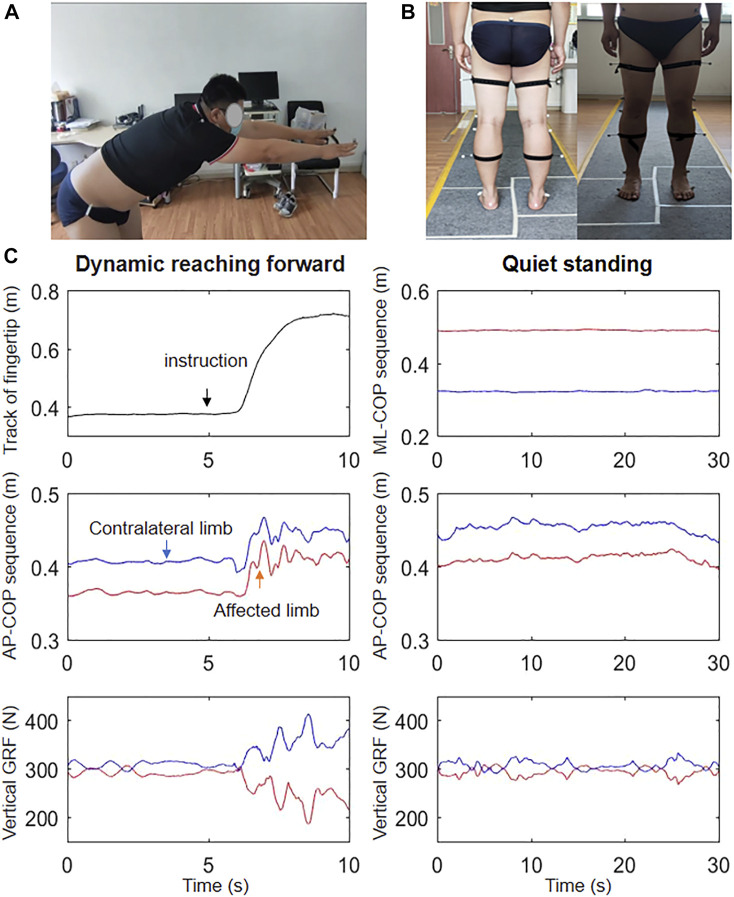
Overview of the **(A)** reaching forward and **(B)** quiet standing tasks performed during the experiment, and **(C)** the tracks of the fingertips, AP-COP, and vertical RFG signals during reaching forward and the ML-COP, AP-COP, and vertical RFG signals during quiet standing in a representative patient.

A three-dimensional motion and capture system (BTS Bioengineering Corp., Italy) with six cameras and two force plates was used during the experiment. The three-dimensional positions of four retro-reflective markers placed on the bilateral shoulders and tips of the middle fingers were recorded at a sampling frequency of 100 Hz (Figure 1A). Two adjacent force plates, spliced together and embedded horizontally into the ground, measured the COP and ground reaction force (GRF) signals at a sampling frequency of 200 Hz. The dynamic signals were synchronous with the kinematic signals.

### 2.3 Test protocol

The IKDC and Lysholm scale values of each patient after ACL reconstruction were acquired by the same trained personnel before testing. For the first task, all subjects conducted standing upright at the specified locations of the two force plates (each foot on a different plate) for 30 s (Figure 1B). For the second task, all subjects stood upright at the identical locations in the first task, while maintaining the bilateral arms and hands outstretched horizontally. The operator gave the order. Subjects reached forward as far as capable without lifting up their heels (Figure 1A). Subjects repeated this process using the right arm, and both arms simultaneously. Two trails were conducted for each task with 2-min interval between trials. Participants were asked to practice the movements several times before measurements.

### 2.4 Data analysis

**FIGURE 2:**
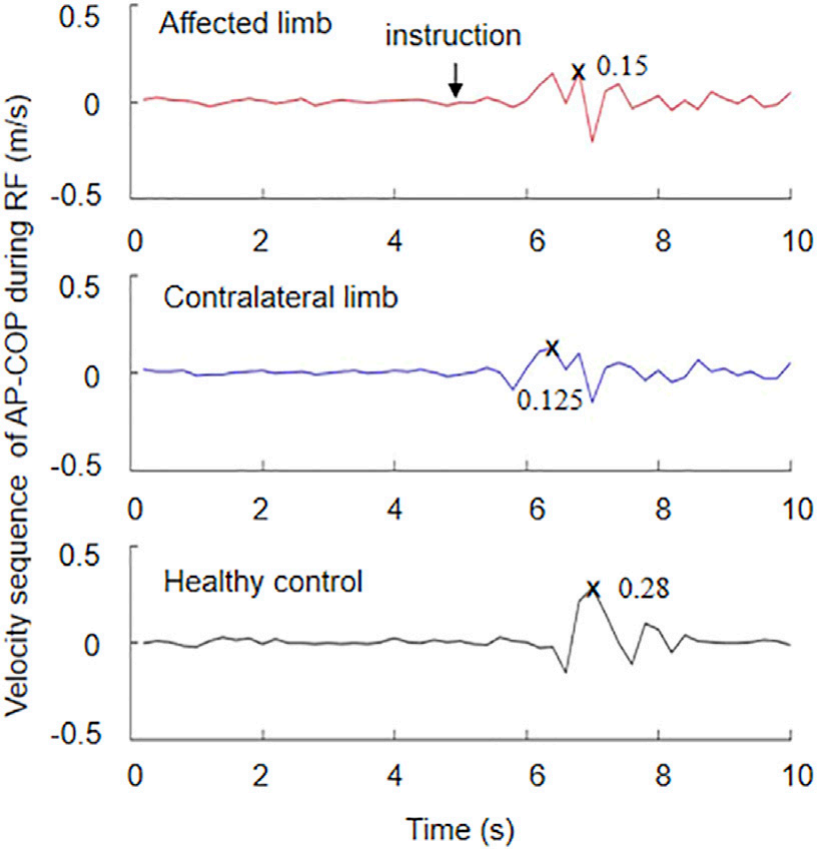
Velocity sequence of AP-COP during reaching forward calculated by a 200 ms slide-window.

Figure 1C depicts the fingertip tracking, AP-COP sequence, and vertical GRF during dynamic forward, as well as the COP in the medial-lateral direction (ML-COP) sequence, AP-COP sequence, and vertical RFG during quiet standing. The subject stood on the force plates stably before the system triggered. The command ‘reach forward’ was made at the 5th second of standing upright with the bilateral arms and hands outstretched horizontally. The MRD was calculated as the difference between the maximal position and the preparation position, i.e., the mean value during the 3–4 s. The speed of forward movement was calculated as the MRD divided by time during reaching forward. The maximal position was defined the peak point during reaching forward. The length of COP was calculated as the difference between the maximum AP-COP and the minimum AP-COP during RF. These parameters were normalized to height. The velocity sequence of AP-COP during RF was calculated by a 200 ms slide-window (Figure 2). The peak speed was the maximum value of the velocity sequence. The weight bearing ratio (WBR) on the affected side was defined as the GRF of the affected limb divided by the total GRF. The MATLAB 2016a (The Mathworks, Natick, MA, USA) was used for the data analysis.

The amplitude of COP variability was quantified using the formula for standard deviation (SD), as follows:

$$SD=\sqrt{\frac{1}{n}\sum_{i=1}^{n}{\left(x\left(i\right)-\bar{x}\right)}^{2}}$$ (1)

where *x*(*i*) is the magnitude of the COP signal at each time point *i,* $\bar{x}$ is the mean value of the *x*(*i*), and *n* is the signal length of *x*(*i*).

The velocity sequence (V) of COP during quiet standing was defined by the equation:

$$V\left(i\right)=\frac{\sqrt{{\left(x\left(i+1\right)-x\left(i\right)\right)}^{2}+{\left(y\left(i+1\right)-y\left(i\right)\right)}^{2}}}{\Delta t}$$ (2)

where *x*(*i*) and *y*(*i*) are the magnitude of the AP-COP and ML-COP signals at each time point *i,* Δt is the time between two adjacent sampling points. The mean and peak speeds of COP during quiet standing was the mean and peak values of the COP velocity sequence.

The power spectral density (PSD) of the COP sequence was calculated to analyze the low-frequency (LF: less than 1 Hz) and high-frequency (HF: 1–10 Hz) components of COP signal. The COP had been filtered with a cutoff frequency of 15 Hz by a Butterworth low-pass filter.

Statistical analyses were performed by SPSS 20.0 (SPSS Inc., Chicago, IL). All variables were verified as normal distribution by the Kolmogorov-Smirnov test. Independent *t*-tests were applied to examine the differences between patient group and control group, patients’ affected limbs and healthy limbs, patients’ contralateral limbs and healthy limbs. The healthy limbs of the controls were corresponding to the affected limbs of the paired patients. For example, the patient’s affected limb is left limb, then the left limb of the healthy subject was selected as the healthy limb. Paired *t*-tests were used to examine the difference between the affected limb and the contralateral limb. No interaction was considered across these *t*-tests. Similarly, bivariate correlation analysis was performed to assess the correlations between the IKDC/Lysholm scales and variables during RF and quiet standing. Values were considered statistically significant at *p* < 0.05.

## 3 Results

**FIGURE 3:**
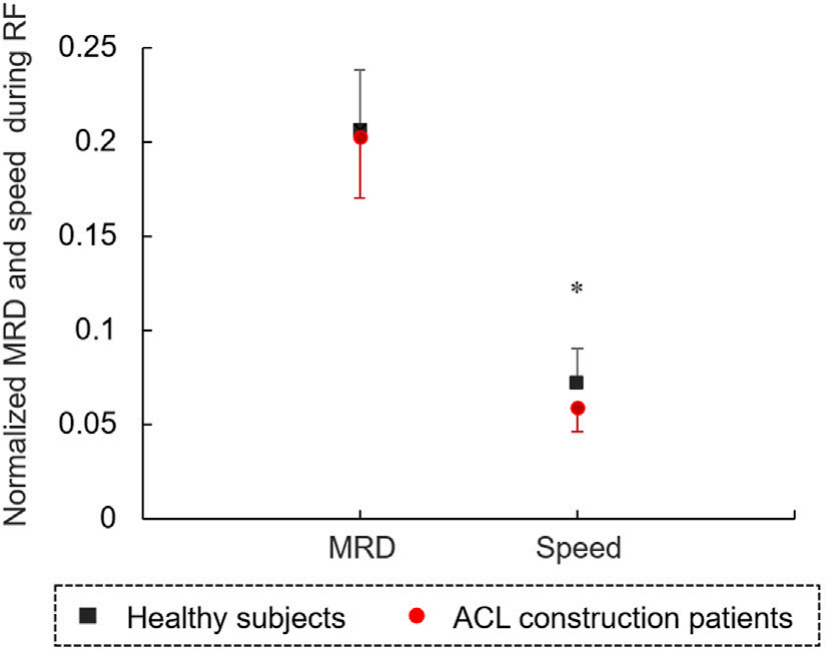
The MRD and speed of forward movement, normalized by height during reaching forward.

The characteristics of the 14 patients with ACL reconstruction (5 women and 9 men; aged 27.74 ± 7.27 years) and 14 healthy controls (5 women and 9 men; aged 27.07 ± 6.65 years) are shown in Table 1. There was no significant difference in age (t = 0.026, *p* = 0.979), height (t = 1.485, *p* = 0.150) and weight (t = 1.851, *p* = 0.078) between two groups. The results of the MRD and speed normalized by height during reaching forward are shown in Figure 3. The speed of RF in the patients with ACL reconstruction normalized by height was significantly lower than that of controls (t = −2.983, *p* < 0.05). No significant difference was observed in the MRD value between the patients with ACL reconstruction and controls (*p* > 0.05).

**TABLE 1:** Descriptive characteristics of subjects.

Number		ACL reconstruction patients (n = 14)						Control (n = 14)			
	Gender	Age(years)	Affected side	Height(cm)	Weight(kg)	IKDC	Lysholm	Gender	Age(years)	Height(cm)	Weight(kg)
1	M	30	R	170	69	-	-	M	31	170	80
2	F	19	L	185	85	94.25	96	F	21	168	54
3	F	22	L	178	73	-	-	F	21	164	52.5
4	M	29	R	186	70	70.11	75	M	30	170	87
5	F	29	L	161	68.5	70.11	95	F	29	160	60
6	M	19	R	175	88	77.01	69	M	19	181	75
7	F	32	R	165	62	62.07	59	F	31	162	58
8	M	18	L	175	75	48.28	23	M	19	168	60
9	F	32	L	161	51	39.08	58	F	32	167	60
10	M	41	L	181	83	95.4	91	M	39	168	64
11	M	26	R	173	110	95.4	65	M	24	180	85
12	M	24	R	193	80	90.8	100	M	23	172	65
13	M	40	R	168	63	81.61	89	M	39	170	63
14	M	19	L	183	92	85.06	89	M	21	175	95

The n is the number of subjects.

**FIGURE 4:**
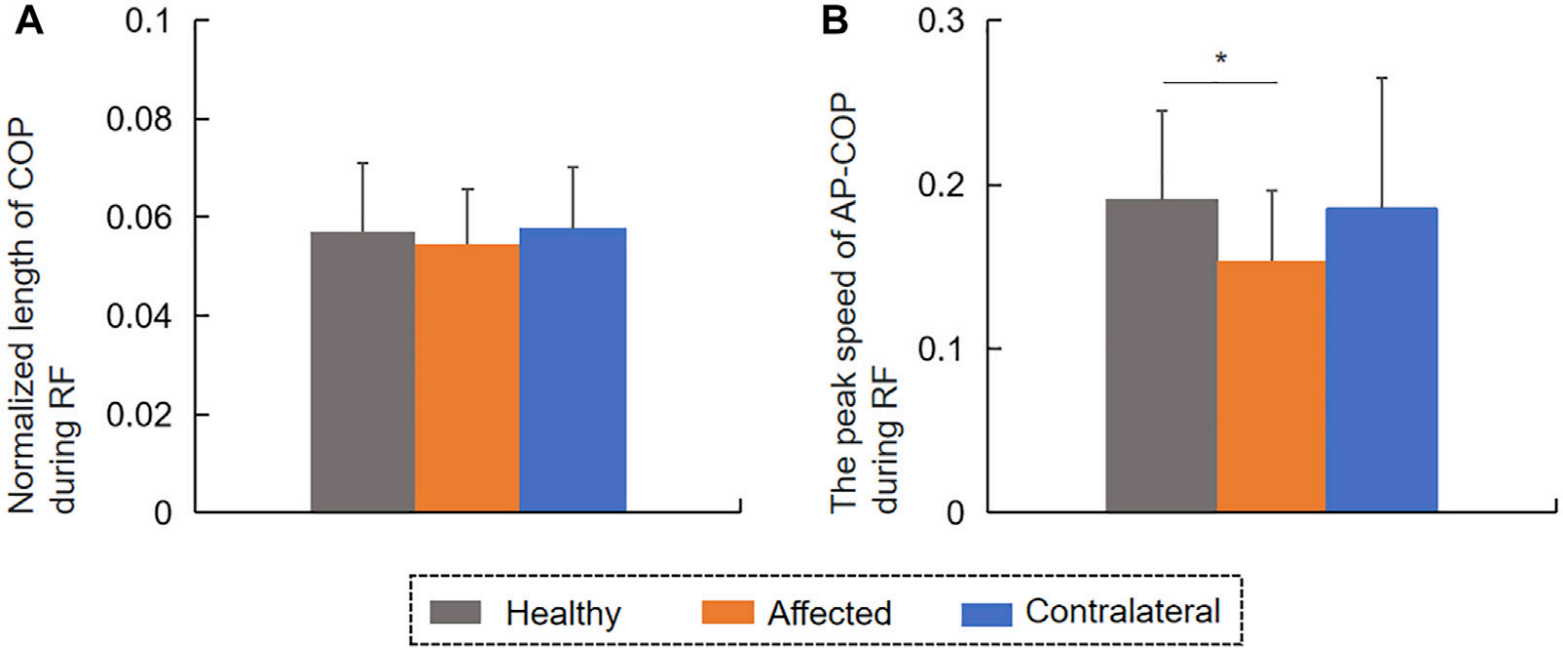
The **(A)** COP length normalized by height and the **(B)** peak speed of AP-COP during reaching forward.

**FIGURE 5:**
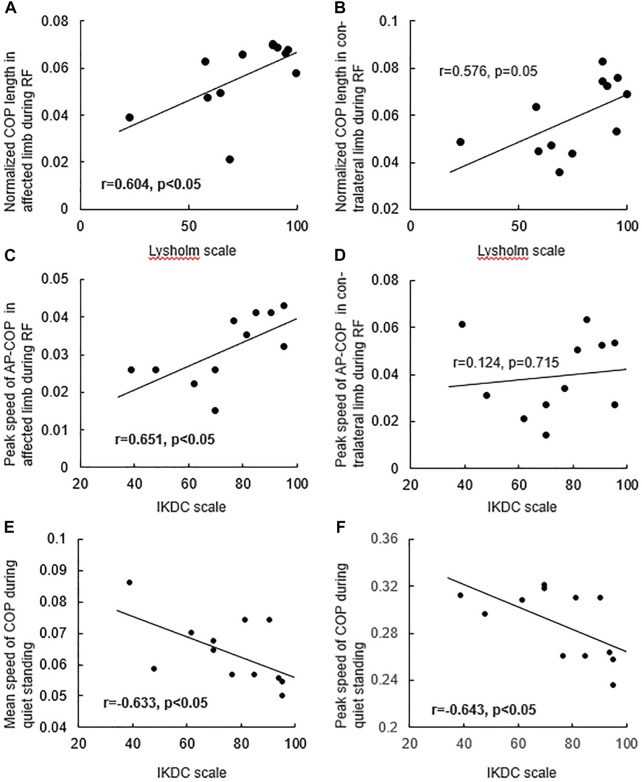
The relationship between the COP length normalized by height in **(A)** affected limb, **(B)** contralateral limb and Lysholm scale during reaching forward, and the relationship between the peak speed of AP-COP in **(C)** affected limb, **(D)** contralateral limb and IKDC scale during reaching forward, and the relationship between the **(E)** mean speed, **(F)** peak speed of COP and IKDC scale during quiet standing.

No significant difference was found in the COP length normalized by height between affected and healthy limbs, or between contralateral and healthy limbs, or between affected and contralateral limbs during RF (*p* > 0.05, Figure 4A). The peak speed of the AP-COP in the patients’ affected limb was significantly lower than that of healthy limb during RF (t = −2.279, *p* < 0.05, Figure 4B). The normalized COP length in the patients with ACL reconstruction was positively correlated with the Lysholm scale in affected limb (r = 0.604, *p* < 0.05, Figure 5A), instead of the contralateral limb (r = 0.576, *p* = 0.05) (Figure 5B) during RF. The peak speed of AP-COP in the patients’ affected limb was positively correlated with the IKDC scale (r = 0.651, *p* < 0.05) (Figure 5C); however, no significant correlation was found between AP-COP and the IKDC scale in the contralateral limb (r = 0.124, *p* = 0.715).

**FIGURE 6:**
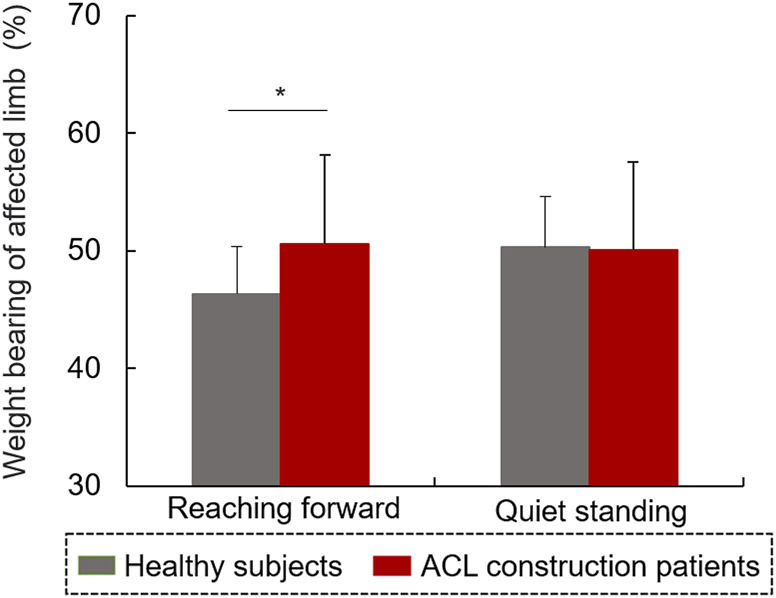
Weight bearing of the affected limb during the reaching forward task and static standing.

The WBR on the affected side in patients with ACL reconstruction was significantly lower than that (50.61%) in the controls during RF (t = −2.17, *p* < 0.05, Figure 6). There was no significant difference in the WBR on the affected limb between the two groups during RF (*p* > 0.05).

**FIGURE 7:**
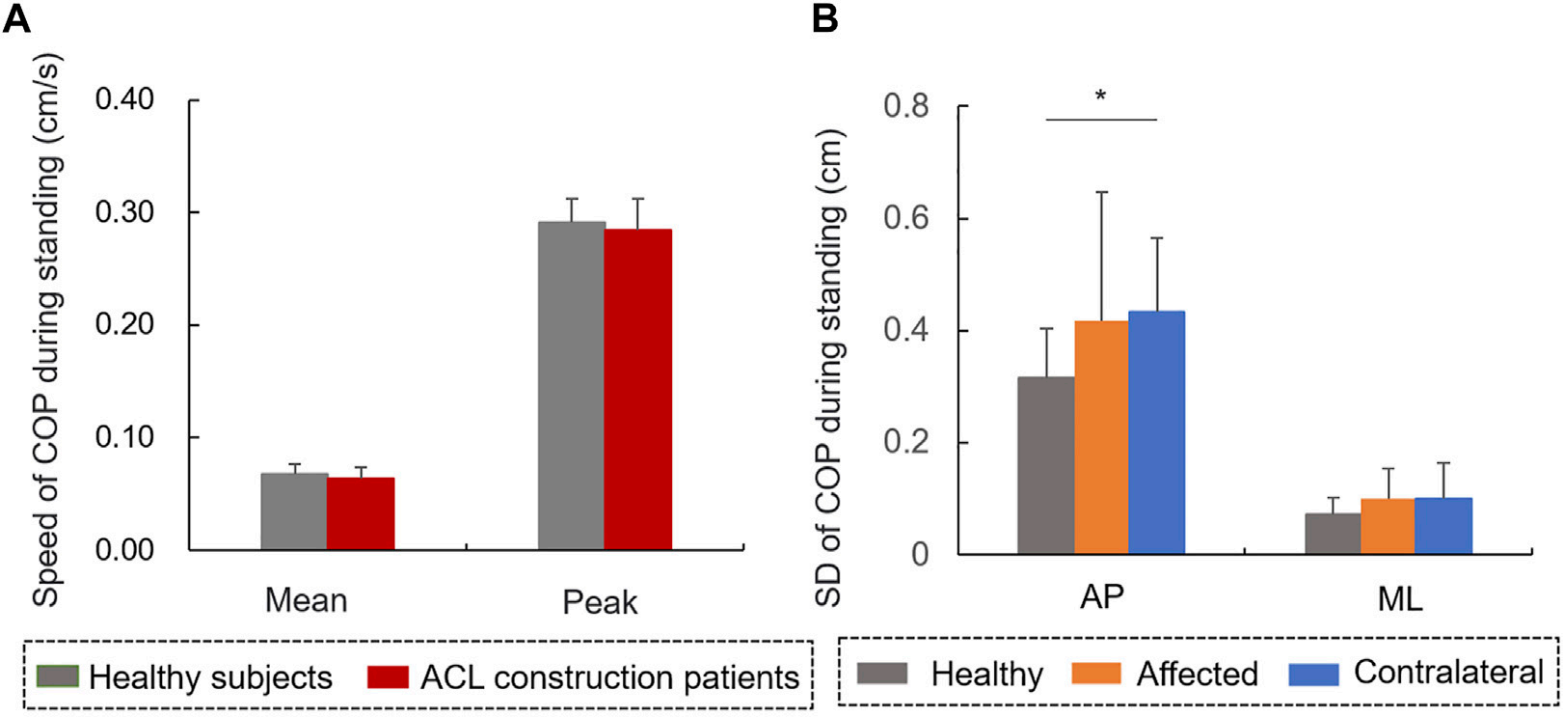
The **(A)** speed of COP trajectory and **(B)** amplitude of COP during quiet standing.

No significant differences were observed in terms of mean or peak speed between patients with ACL reconstruction and controls during quiet standing (Figure 7A). The amplitude of AP-COP variability in contralateral limb was significantly higher than that in healthy limb during standing (t = 2.196, *p* < 0.05, Figure 7B). No significant differences were found in the amplitude of ML-COP variability between affected and healthy limbs, or between contralateral and healthy limbs, or between affected and contralateral limbs. The mean (r = −0.633, *p* < 0.05, Figure 5E) and peak speed (r = −0.643, *p* < 0.05, Figure 5F) values were negatively correlated with the IKDC scale values.

**FIGURE 8:**
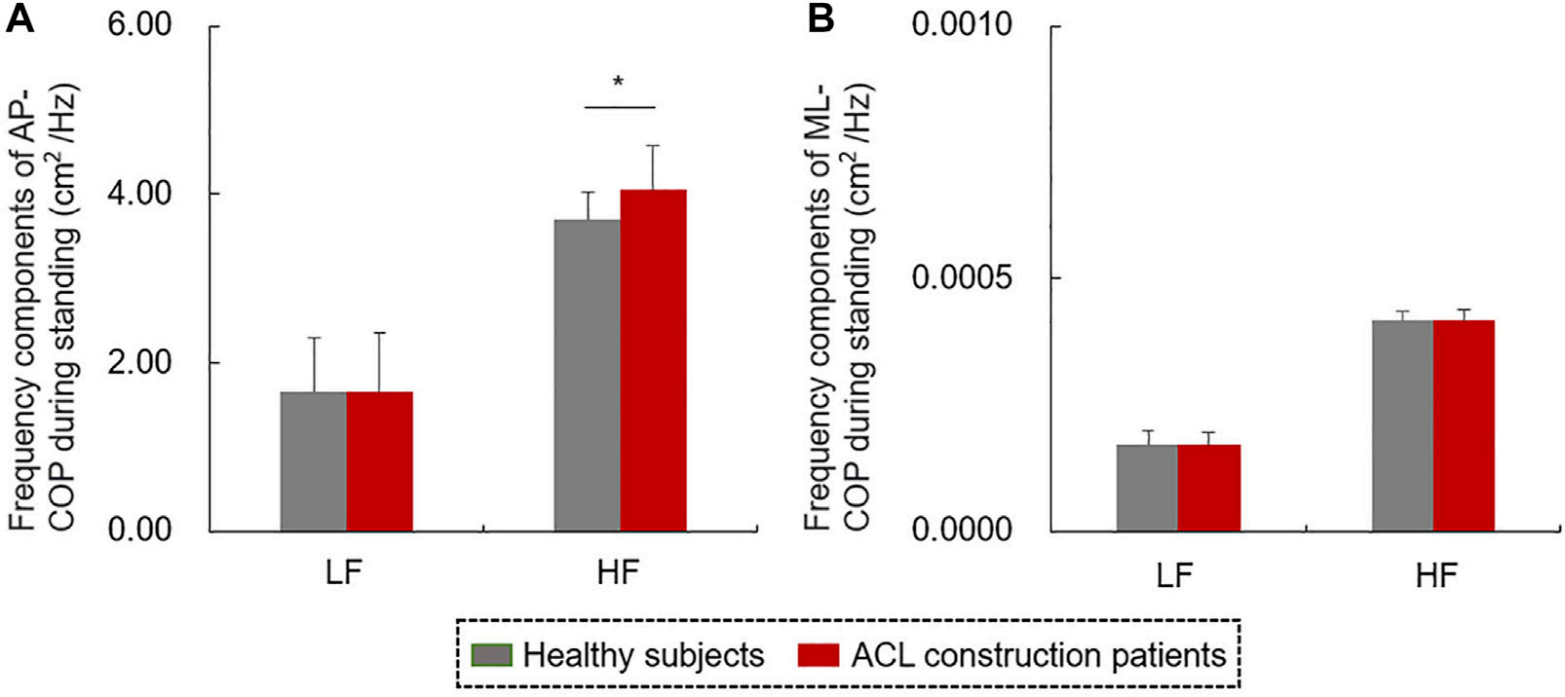
The frequency components of **(A)** AP-COP and **(B)** ML-COP signals during quiet standing.

The frequency components of COP signals during quiet standing were shown in Figure 8. The HF component of AP-COP in patients with ACL reconstruction was significantly higher than that of healthy subjects (t = 2.074, *p* < 0.05, Figure 8A). There was no significant difference in HF component of ML-COP, LF component of AP-COP or ML-COP between patients and healthy subjects.

## 4 Discussion

This study investigated the balance control capacity during static and dynamic conditions of patients after ACL reconstruction. The patients with ACL reconstruction showed a lower speed of RF compared with controls, although no difference was observed in the MRD values between the two groups. A reduced peak speed of the affected AP-COP indicates an inadequate control ability of COP transfer during dynamic balance condition in patients with ACL construction. In our study, the patients’ affected limbs bore less weight only during dynamic RF instead of quiet standing. Patients with ACL reconstruction further showed a higher amplitude of COP variability and HF component of COP in the AP direction compared with controls during standing. In the dynamic RF condition, COP antedisplacement value was positively correlated with the Lysholm scale value. In the static standing condition, the values of mean and peak speeds of COP were negatively correlated with the values of IKDC scale. Overall, all of these results contribute to our understanding of patients’ balance function 6 months after ACL reconstruction, and will help in formulating targeted strategies for rehabilitation training in clinical settings.

COP amplitude variability quantified by SD is considered to be an indicator of balance control and postural stability (Rakhra & Singer, 2022). The increased amplitude variability of AP-COP in the contralateral side reflects compensation of the contralateral limb and enhancive postural sway after ACL reconstruction during quiet standing (Yu et al., 2008). It has been reported that the LF and HF components of COP reflect the sensitivity of vestibular and visual information, and somatosensory inputs including proprioception (Kouzaki & Masani, 2012). Increased HF component of AP-COP indicated the reduced proprioception in patients with ACL reconstruction (Mcchesney & Woollacott, 2000; Kouzaki & Masani, 2012). No similar results were found in ML-COP variability, which indicates that the balance control in the AP, but not the ML direction, was influenced by ACL injury. In addition, no significant differences were found in terms of mean or peak speed of COP trajectory, or the WBR of affected limb between patients with ACL-reconstruction and controls during quiet standing. These findings suggested that patients had partly regained their static balance control ability 6 months after ACL reconstruction. It has been reported that a higher COP speed indicates increased postural instability and falling risk (Yilmaz & Bulbuloglu, 2021). In the current study, both the mean and peak speed values of COP trajectory during quiet standing were negatively correlated with the IKDC scale values, indicating that the decrease of knee function after ACL injury, including pain, stiffness, and swelling, causes instability of the static balance control and fast postural adjustment (Bodkin et al., 2018).

The MRD, defined as the distance the fingertip travels while RF, indicates the balance boundary and postural control capacity of the participant (Lin et al., 2010). In the current study, the MRD value in patients with ACL was not different from that of controls, demonstrating that patients had a normal reaching boundary 6 months after ACL reconstruction. However, the reduced speed of RF suggested that these patients still required more time to reach the target distance. This inefficient strategy may expend more energy and could be linked to a lack of confidence and fear of falling.

The lower weight bearing on the affected limb indicated inadequate supporting capacity requiring compensation by the contralateral limb during dynamic balance control after ACL reconstruction. Previous studies have reported that altered nerve reflex pathways may partially inhibit α motoneuron excitability of the quadriceps and increase hamstring muscle activity in patients after ACL reconstruction (Burland et al., 2020; Suh et al., 2021). Additionally, weight bearing is known to be related to quadriceps muscle strength and function in patients with ACL-reconstruction (Chmielewski et al., 2002). Therefore, this finding further implies that the inadequate supporting capacity was possibly contributed by the knee instability, pain, muscle strength weakness, and decreased muscle coordination in 6 months after ACL reconstruction.

COP signals during postural control provide neuromuscular and somatosensory information. A previous study demonstrated that muscle strength and proprioception are significantly associated with postural stability, as represented by COP variables, in patients with unilateral knee osteoarthritis (Zeng et al., 2022). In the present study, the reduced peak speed of AP-COP of the affected limb during RF suggested that ACL reconstruction reduced the control ability of COP transfer and changed the dynamic balance control strategy (Bulow et al., 2021). In addition, the peak speed of AP-COP of the affected limb positively correlated with the IKDC scale. These findings suggested that deficient knee function limited the forward transfer speed of COP. Although the COP antedisplacement of patients with ACL reconstruction did not differ from that of controls during RF, the antedisplacement value of COP positively correlated with the Lysholm scale value. This result implies that the narrowed equilibrium boundary during RF may be partly generated by knee instability, pain, and lock following ACL reconstruction. The peak speed of AP-COP during RF could be considered as an evaluation indicator for patients following ACL construction in clinical practice. In clinical rehabilitation, balance control and proprioception training, such as vertical vibration and single-leg standing with eyes closed, supporting capacity training of affected limb should be considered to improve balance function (Faggal et al., 2019).

This study had some limitations. First, the sample size was too small to fully reveal the relationship between the ACL reconstruction and balance control ability. Second, we did not record surface EMG signals, or behavioral, and clinical outcomes. Further studies are needed to address these gaps. Third, this is a single center study.

## 5 Conclusion

At 6 months after surgery, patients with ACL reconstruction regained part of their balance ability, but showed decreased postural control and loading ability of the affected limb, especially in terms of dynamic balance, suggesting the adoption of an abnormal postural control strategy. The COP length, peak speed during RF and COP speed during quiet standing could be considered as quantitative assessment index for balance function of patients with ACL reconstruction. Clinicians should be aware of the dynamic balance training, correcting postural control strategy, and improving supporting capacity in affected limb should be a primary goal during rehabilitation following ACL reconstruction.

## Data Availability

The original contributions presented in the study are included in the article/Supplementary Material, further inquiries can be directed to the corresponding author.
